# Serum endostatin levels are associated with diffusion capacity and with tuberous sclerosis- associated lymphangioleiomyomatosis

**DOI:** 10.1186/s13023-019-1050-4

**Published:** 2019-03-29

**Authors:** Anthony M. Lamattina, Sergio Poli, Pranav Kidambi, Shefali Bagwe, Andrew Courtwright, Pierce H. Louis, Shikshya Shrestha, Benjamin Stump, Hilary J. Goldberg, Elizabeth A. Thiele, Ivan Rosas, Elizabeth P. Henske, Souheil El-Chemaly

**Affiliations:** 1 Division of Pulmonary and Critical Care Medicine, Brigham and Women’s Hospital, Harvard Medical School, Boston, MA 02115 USA; 2 Division of Pulmonary and Critical Care Medicine, Hospital of the University of Pennsylvania, Philadelphia, PA USA; 3 Department of Neurology, Massachusetts General Hospital, Harvard Medical School, Boston, MA USA

**Keywords:** LAM, TSC, Endostatin, DLCO, Isolated decrease

## Abstract

Endostatin is a naturally occurring collagen fragment with anti-angiogenic properties. We investigated the association between serum endostatin levels and DLCO in a cohort of patients with lymphangioleiomyomatosis (LAM). Associations of endostatin levels to clinical features of LAM were explored using logistic regression models. Endostatin levels were associated with DLCO and were higher in subjects with TSC-associated LAM compared to sporadic LAM. These data suggest that endostatin could be a predictive biomarker of decline in DLCO and that germline mutational inactivation of the TSC1 or TSC2 gene is associated with higher endostatin levels. These findings could offer novel insights into the pathogenesis of LAM.

## Introduction

Lymphangioleiomyomatosis (LAM) is a multisystem disease characterized by mutations in the tuberous sclerosis complex (TSC) genes. LAM almost exclusively affects women and can occur sporadically (sporadic LAM) or in association with TSC (TSC-LAM). Angiomyolipomas (AML), which are benign blood-vessel-filled kidney tumors, occur in approximately one-third of women with sporadic LAM and in the majority of women with TSC-LAM. Vascular endothelial growth factor (VEGF)-D is a biomarker for LAM and is significantly elevated in both sporadic LAM and TSC-LAM compared to healthy controls [1]. We have recently shown that a subgroup of patients with LAM can present with an isolated reduction in diffusion capacity of the lungs for carbon monoxide (DLCO) that does not correlate with evidence of pulmonary hypertension or VEGF-D levels [2]. Endostatin, a cleavage product of collagen XVIII, alpha 1 (Col18a1), is a potent inhibitor of angiogenesis in vitro and in vivo [3, 4]. Interestingly, endostatin levels correlate with DLCO in scleroderma and mixed connective tissue disease [5]. We hypothesized that endostatin levels are altered in patients with LAM and correlate with DLCO.

## Methods

Women with LAM and healthy female controls (recruited through local advertisement) were enrolled at Brigham and Women’s Hospital in IRB-approved protocols (IRB 2008P002027 and 2012P000840). LAM and TSC were diagnosed using established criteria [6, 7]. All subjects were non-smokers and all patients with LAM in this analysis were not receiving therapy with mammalian/ mechanistic target of rapamycin inhibitors. Serum samples were collected from each subject using standardized protocols. Data on age, presence of AML, and pulmonary function tests (PFT) were collected from the medical records. PFT were performed in a clinical laboratory according to published standards. Of the 58 subjects with PFT values, 29 (50%) had measurements recorded on the same day as sample collection, while the average time between PFT measurement and sample collection for all subjects was 42.1 ± 14.5 days (mean ± SEM). Endostatin serum concentrations were determined with an enzyme-linked immunosorbent assay (ELISA; R&D systems, Minneapolis) as per manufacturer’s instructions.

Receiver operating characteristics (ROC) curves were generated to determine if endostatin levels were effective in identifying women with TSC-LAM versus sporadic LAM. We subsequently calculated the area under the curve (AUC) and corresponding 95% confidence interval by the DeLong method, and the optimal endostatin cutpoint value to differentiate TSC-LAM and sporadic LAM subjects was determined using the Youden method [8].

Isolated reduction in DLCO was defined as subjects with FEV1 and FVC > 80% predicted and DLCO < 60% predicted. Other variables included in analyses were age as a continuous variable and angiomyolipoma (AML) status (presence or absence). To evaluate endostatin concentration by diagnosis, subjects with sporadic LAM, TSC-LAM and healthy controls were included in the analysis. Baseline characteristics were compared using a two-sample Student’s T-test, Mann-Whitney U test, or Kruskal-Wallis *H* test as necessary. Fisher’s Exact tests were employed for binary variables. Pairwise comparisons of endostatin level for each diagnosis group were performed using a Dunn test with *p* values adjusted via the Benjamini-Hochberg method. Logistic regression models were utilized to assess univariate associations between TSC-LAM versus sporadic LAM and variables of interest. Linear regression models were implemented to determine predictors of serum endostatin concentration. Multivariate regression models for outcomes of interest contained all significant predictors from the respective univariate models. All statistical analyses were completed using R (version 3.5.1) [9].

## Results

**Fig. 1:**
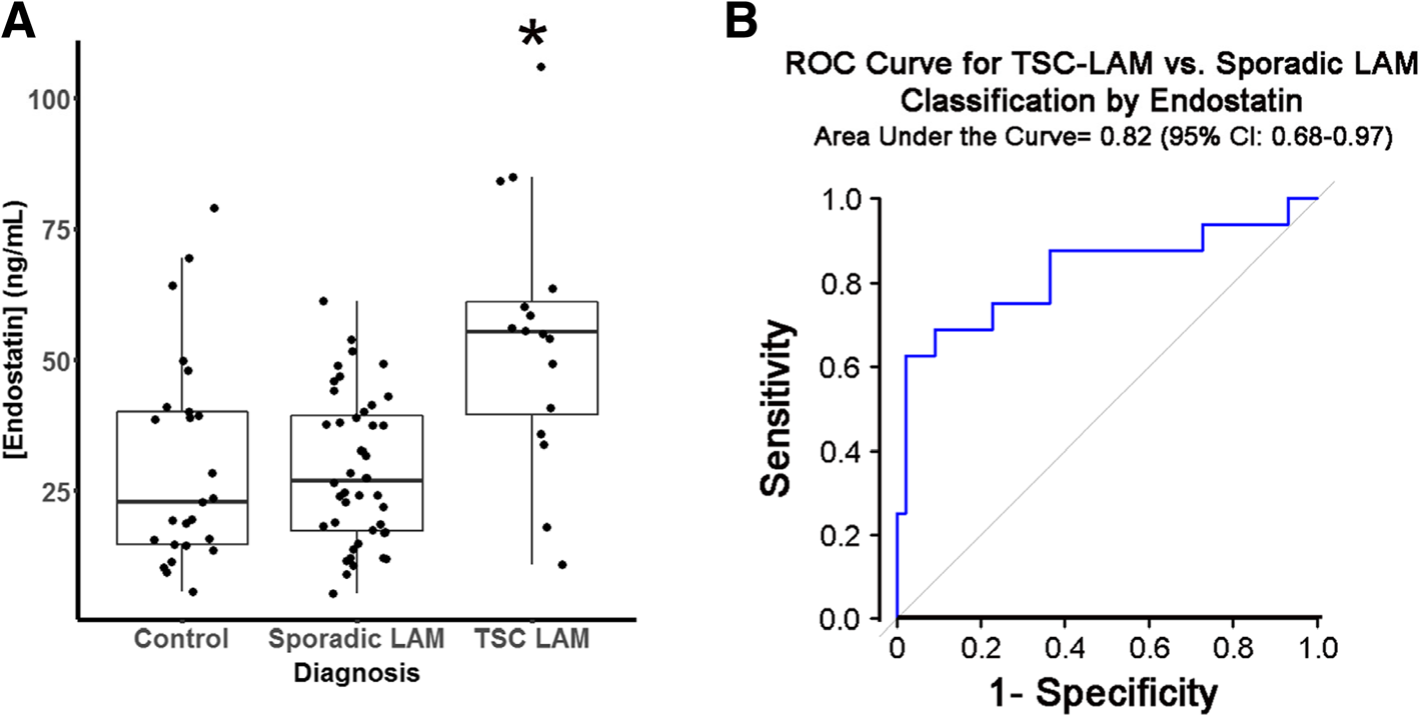
Endostatin concentrations differentiate TSC-LAM and sporadic LAM. **a** Boxplots of endostatin serum concentration per diagnosis (* *p* < 0.001 compared to control and Sporadic-LAM). **b** Receiver operator curve (ROC) for predicting TSC-LAM versus sporadic LAM by endostatin serum concentration

In univariate analysis of serum endostatin levels, we found that DLCO (β − 0.28; 95% CI -0.55, − 0.016; *p* < 0.05), isolated reduction in DLCO (β 24.1; 95% CI 8.6, 39.6; *p* < 0.01) and the presence of AML (β 10.8; 95% CI 0.31, 21.2; *p* < 0.05) correlated with endostatin levels. Importantly, while there was no difference in endostatin level comparing sporadic LAM and controls (β − 1.2; 95% CI -10.2, 7.9; *p* = 0.80), we found an association between endostatin levels and TSC-LAM versus controls (β 24.1; 95% CI 12.5, 35.7; *p* < 0.001) (Table 1, Fig. 1a). Furthermore, a ROC curve assessing endostatin level’s efficiency in discriminating TSC-LAM from sporadic LAM produced an AUC of 0.82 (95% CI 0.68, 0.97; *p* < 0.001) and an optimal endostatin cutpoint of 54.2 ng/ml (Fig. 1b). To further investigate this relationship, we examined the baseline characteristics of controls and subjects with sporadic LAM and TSC-LAM. Sporadic LAM subjects were significantly older than healthy controls (*p* < 0.01), but age did not reach statistical significance in any other paired comparison. TSC-LAM subjects were more likely to have an AML, and had higher endostatin levels than subjects with Sporadic LAM (Table 2). Because there were no TSC-LAM patients without AMLs, we could not include AML as a predictor in any logistic regression models. In univariate logistic models, age (OR 0.56 for 10-year increase; 95% CI 0.31, 0.93; *p* < 0.05), isolated reduction in DLCO (OR 8.86; 95% CI 1.67, 68.17; p < 0.05) and endostatin levels (OR 2.22 for 10 ng/mL increase; 95% CI 1.48, 3.82; *p* < 0.001) were predictive of TSC status (Table 3). In a multivariate model, only endostatin level (OR 2.51 for 10 ng/mL increase; 95% CI 1.53, 4.87; *p* < 0.01) was a significant predictor of TSC-LAM (Table 3).

**Table 1:** Associations of serum endostatin concentration with patient characteristics

Variable	N	Mean Serum [Endostatin] (ng/mL) (SE)	Beta Estimate (95% CI)	P
Diagnosis^a^				
Control (Reference)	25	30.1 (4.0)		
Sporadic LAM	44	28.9 (2.1)	−1.2 (− 10.2, 7.9)	0.80
TSC LAM	16	54.2 (6.1)	24.1 (12.5, 35.7)	0.00008
Age (years)^a^	84	–	0.15 (− 0.18, 0.48)	0.36
FEV1 (%)^b^	58	–	−0.011 (− 0.27, 0.25)	0.93
FVC (%)^b^	58	–	0.018 (− 0.29, 0.33)	0.91
DLCO (%)^b^	57	–	−0.28 (− 0.55, − 0.016)	0.038
Isolated Reduction in DLCO (< 60%) ^b^				
No (Reference)	50	31.8 (2.4)		0.0029
Yes	7	55.9 (11.9)	24.1 (8.6, 39.6)	
AML^b^				
No (Reference)	25	29.4 (2.8)		0.044
Yes	35	40.1 (4.0)	10.8 (0.31, 21.2)	

^a^Model includes all LAM patients and Control subjects

^b^Model includes only LAM patients

**Table 2:** Variable comparison for TSC LAM versus Sporadic LAM diagnosis

Variable	TSC *N* = 16	Sporadic *N* = 44	Control *N* = 25	P
Age (years) (mean ± SE)	41.6 ± 2.9	49.4 ± 1.8	39.4 ± 3.2	0.003^1^
FEV1 (%) (mean ± SE)^5^	90.9 ± 4.3	86.7 ± 3.6	–	0.46^2^
FVC (%) (mean ± SE)^5^	99.6 ± 3.7	98.1 ± 3.0	–	0.75^2^
DLCO (%) (mean ± SE)^6^	65.9 ± 5.9	67.7 ± 2.9	–	0.68^3^
Endostatin (ng/mL) (mean ± SE)	54.2 ± 6.1	28.9 ± 2.1	30.1 ± 4.0	0.0007^1^
AML				
No, n (%)	0 (0)	25 (56.8)	–	0.00005^4^
Yes, n (%)	16 (100)	19 (43.2)		
Isolated Reduction in DLCO (< 60%) ^6^				
No, n (%)	11 (68.8)	39 (95.1)	–	0.015^4^
Yes, n (%)	5 (31.2)	2 (4.9)		

^1^ Kruskal-Wallis *H* test

^2^ Two-sample T test with unequal variance

^3^ Mann-Whitney *U* test

^4^ Fisher’s Exact Test

^5^42 Sporadic LAM patients in analysis

^6^41 Sporadic LAM patients in analysis

**Table 3:** Associations of TSC versus Sporadic LAM with subject characteristics

	Unadjusted OR (95% CI)	P value	Adjusted OR (95% CI)	*P* value
Age (10-year increase)				
Sporadic	1.00	0.035	1.00	0.086
TSC	0.56 (0.31, 0.93)		0.54 (0.25, 1.04)	
Endostatin (10-ng/mL increase)				
Sporadic	1.00	0.0008	1.00	0.0015
TSC	2.22 (1.48, 3.82)		2.51 (1.53, 4.87)	
Isolated Reduction in DLCO				
Sporadic	1.00	0.016	1.00	0.29
TSC	8.86 (1.67, 68.17)		3.95 (0.33, 58.42)	

Odds ratio associations were calculated via logistic regression models. Adjusted ORs were computed in models featuring age, serum endostatin concentration, AML, and isolated reduction in DLCO as covariates

In a separate analysis conducted to evaluate the relationship between AML and endostatin levels, we found that there was no difference in endostatin levels between sporadic LAM subjects with (28.3 ± 3.4; *N* = 19) and without AML (29.4 ± 2.8; *N* = 25) (*p* = 0.82). Furthermore, subjects with TSC and AML (54.2 ± 6.1; *N* = 16) had higher serum endostatin levels than those with sporadic LAM and AML (*p* < 0.01).

## Discussion

These data show that endostatin levels are associated with DLCO in a cohort of patients with LAM. Surprisingly, isolated reduction in DLCO is associated with TSC status. These findings are novel and may point toward unexpected mechanistic differences in disease pathogenesis between sporadic LAM and TSC-LAM. Many biomarkers have previously been described in LAM [10], but none to our knowledge would differentiate between TSC-LAM and sporadic LAM.

We hypothesize that the association of endostatin with DLCO impairment may be a consequence of its anti-angiogeneic activity and resulting decrease in lung vasculature. However, we have previously shown that in this population, isolated reduction in DLCO does not correlate with surrogate markers of pulmonary hypertension [2]. Pre-clinical studies examining the role of endostatin in disease initiation and progression are needed.

The source of elevated endostatin levels in TSC is unknown. AMLs could be a potential source since they are more common in TSC. However, our analysis suggests that serum endostatin levels are independent predictors of TSC. Moreover, the presence of AML did not appear to be associated with endostatin levels in sporadic LAM. An analysis of the tissue atlas database www.proteinatlas.org) showed that Col8a1 RNA expression is highest in female reproductive organs and in the liver [11]. Intriguingly, matrix metalloproteinase 9 (MMP9), which is known to be dysregulated in LAM [12], has been shown to increase endostatin production in breast cancer cells [13].

In summary, we found that serum endostatin levels were associated with DLCO and with the diagnosis of TSC-LAM in comparison to sporadic LAM. Additional studies are needed to validate these findings and to study the importance of endostatin in disease pathogenesis. In particular, it will be important to determine whether endostatin levels are elevated in other phenotypes of TSC, including individuals with AML who do not have LAM.

## Abbreviations

AML: Angiomyolipoma
DLCO: Diffusing Capacity of the Lung for Carbon Monoxide
LAM: Lymphangioleiomyomatosis
TSC: Tuberous Sclerosis Complex
VEGF-D: Vascular Endothelial Growth Factor- D
